# Is social participation associated with good self-rated health among visually impaired older adults?: the JAGES cross-sectional study

**DOI:** 10.1186/s12877-021-02554-7

**Published:** 2021-10-23

**Authors:** Atsuhide Takesue, Yoshimune Hiratsuka, Akira Inoue, Katsunori Kondo, Akira Murakami, Jun Aida

**Affiliations:** 1 Department of Ophthalmology, Juntendo Nerima Hospital, Tokyo, Japan; 2 Department of Ophthalmology, Juntendo University Graduate School of Medicine, 3-1-3 Hongo, Bunkyo-ku, Tokyo, 113-8431 Japan; 3 Department of Social Preventive Medical Sciences, Center for Preventive Medical Sciences, Chiba University, Chiba, Japan; 4 Department of Gerontological Evaluation, Center for Gerontology and Social Science, National Center for Geriatrics and Gerontology, Obu City, Aichi Japan; 5 Department of Oral Health Promotion, Graduate School of Medical and Dental Sciences, Tokyo Medical and Dental University, Tokyo, Japan; 6 Division for Regional Community Development, Liaison Center for Innovative Dentistry, Graduate School of Dentistry, Tohoku University, Sendai, Japan

**Keywords:** Social participation, Self-rated health, Visual impairment, Interaction analyses, Older adults

## Abstract

**Background:**

While it has been recognized that visual impairment is associated with poor self-rated health (SRH), in addition to various negative health outcomes of visual impairment, the number of older adults with visual impairment is increasing due to population aging. As increasing evidence has been found for the effectiveness of social participation on good SRH, we examined whether there was an association between social participation and SRH and investigated whether the effect differed by visual status.

**Methods:**

Questionnaire data on self-reported visual status, social participation, socioeconomic status, and SRH were obtained in 2016. A total of 24,313 community-dwelling individuals aged 65 and over participated. We examined the association of social participation and SRH status among older adults with visual impairment. Stratified analysis and analysis with an interaction term between social participation and visual status were also conducted. Social participation was assessed by the number of participating groups (no participation, one, two, and three or more).

**Results:**

Overall visual impairment prevalence was 9.3% (95% CI: 8.9–9.7). Among those with and without visual impairment, prevalence of poor SRH was 38.4 and 13.1%, respectively. However, the association between social participation with SRH was similar, especially for those who participated in one or two groups. For people with (PR = 0.54) and without visual impairment (PR = 0.50), those who participated in two groups showed lower prevalence ratios for poor SRH compared to people without social participation.

**Conclusion:**

Social participation showed a beneficial association with SRH among older adults with visual impairment. Future interventions could focus on the potentially positive role of social participation on SRH among older adults with visual impairment.

**Supplementary Information:**

The online version contains supplementary material available at 10.1186/s12877-021-02554-7.

## Background

Population-based studies show that the increase in eye diseases and visual impairment is driven by population aging [1]. About 50% of individuals with visual impairment are older than 70 years, and this percentage is projected to increase [2]. Visual impairment is associated with a wide range of adverse physical and psychological outcomes, such as difficulties in activities of daily living (ADL) [3], and compromised mobility [4], as well as increased reliance on community support services or help from family and friends [5]. This imposes a substantial burden on society [6]. Visual impairment is also associated with increased risk of falls [7], fractures [8], motor vehicle accidents [9], depression [10], cognitive impairment [11], and increased mortality [12]. The association between visual status and poor self-rated health has also been widely recognized [13]. Because of its validity as a predictor of mortality, regardless of other behavioral, psychosocial, or medical factors, self-rated health is perhaps the most widely adopted health status assessment globally [14].

Several studies showed that self-rated health was strongly influenced by social capital, including individual social participation [15–17]. Social participation is a key determinant of active aging and has a positive effect on physical and mental health among older adults [18]. Previous longitudinal studies reported that social participation, as well as the broader concept of “social capital,” was associated with improved functional status among older adults [19, 20]. Additionally, intervention studies aimed at increasing social participation showed a reduction in the incidence of dementia [21]. A prominent characteristic of social participation is its easy inclusion as a target of health promotion programs by health workers compared with other factors associated with self-rated health, such as culture, language use, marital status, educational background, income, health conditions, and so on [22–24]. This means that encouraging older adults to increase their social participation might be a reasonable intervention for the improvement of older adults’ self-rated health.

However, we previously reported that visual impairment was a potential barrier to social participation for community-dwelling older adults. In the study, we revealed older adults’ visual impairment was significantly associated with reduced participation in sports, hobby, volunteer, study/cultural, and health promotion groups; neighborhood associations; and teaching skills/passing on experiences (*p* for trend< 0.01) [25]. Consequently, two interpretations can be made. First, those with visual impairment may have difficulties leaving their homes and reaching an event site even with an intention to participate in social activities. Second, those with visual impairment may recognize that their poor vision makes it more difficult to enjoy social participation even without transportation problems. The latter interpretation may indirectly imply that social participation’s positive impact on self-rated health is weaker in the visually impaired population. In spite of the growing number of older adults with visual impairment, no prior studies have reported the impact of social participation on self-rated health from this perspective.

Therefore, the aim of the present study is to investigate whether there is an association between social participation and good self-rated health and investigate whether the effect differs by visual status. If older adults’ social participation is shown to have a great effect on self-rated health, whether they are visually impaired or not, this would reinforce the importance of including a social participation program in health promotion strategies.

## Methods

### Participants and dataset

Data were acquired from the Japan Gerontological Evaluation Study (JAGES), an ongoing prospective cohort study of the social determinants of health among adults aged ≥65 years. JAGES panel surveys have previously included a broad range of social determinants, psychological factors, and health behaviors. To date, six waves of questionnaire surveys have been conducted between 2003 and 2019. Our analysis is based on cross-sectional data from the 2016 survey conducted in 39 municipalities between October 2016 and January 2017. Questions regarding visual status were appended for the first time in this wave. One-eighth of the target population (*n* = 34,571) was randomly selected to receive the questionnaire. One-eighth of the target population (*n* = 34,571) was randomly selected to receive the questionnaire. Of 34,571 people invited to participate, 24,268 returned the questionnaires (response rate:70.2%). Among the respondents, 1973 were not eligible because they were certified as needing public long-term care. We also excluded four respondents who did not report their gender. Thus, our analytical sample comprised 22,291 individuals. For respondents who experienced difficulty reading or completing the questionnaire, family or friends were allowed to help.

### Variables

#### Subjective health status

One survey question measured self-rated health: “How is your current health status?” with possible responses being “excellent,” “good,” “fair,” and “poor.” Dichotomization of multi-nominal self-rated health is frequently used in studies and has been validated [26]. Answers of “excellent” and “good” were defined as good subjective health status and “fair” or “poor” as poor subjective health status. The test-retest reliability of self-rated health was shown to be good in a variety of subgroups by age and gender [27]. Further, the criterion-related validity of self-rated health was previously shown to predict mortality [28]. Similar results were also observed among older Japanese adults, regardless of age, health behaviors, marital status, chronic comorbidities, and depression symptoms [14].

#### Visual status

Visual status was measured using one question from the English Longitudinal Study of Aging that was translated to Japanese [29]: “Is your eyesight (using glasses or corrective lens as usual) (1) excellent, (2) very good, (3) good, (4) fair, or (5) poor?” Respondents who chose “fair” or “poor” were defined as visually impaired. Self-reported visual status has previously demonstrated a significant association with objective visual acuity [30].

#### Social participation

Social participation was defined as involvement in any kind of social activity. Respondents were asked how often they participated in sports, hobby, health promotion, study/cultural, and volunteer groups; neighborhood associations; senior citizen clubs; and if they were involved in teaching skills/passing on experiences to others. Participation frequency was assessed as: ≥4 times per week, 2–3 times per week, once a week, 1–3 times per month, several times per year, or never. We defined “social participation” as participating in a group with a frequency of at least several times per year. We generated a total participation score to assess the intensity of overall social participation. The total number of group types in which each participant took part was tallied, and participation was categorized from 0 (no participation) to 8 (full participation).

#### Covariates

Various factors have been reported to be associated with subjective health status [22–24], and the potential confounders were included in our analysis. Demographic factors included age (65–69, 70–74, 75–79, 80–84, and ≥ 85 years old) and sex (male/female). Socioeconomic status (SES) included annual equivalized income level (less than 2 million yen = “low”, 2-3.99 million yen = “middle”, and 4 million yen or more = “high”) and years of education (< 9 years, 10 to 12 years, and ≥ 13 years). Social life included marital status (married, widowed, separated, and unmarried). Medical conditions were addressed in the questionnaire as they are associated with both subjective health status and visual status. Respondents were asked whether they had a history of systemic comorbidities, including diabetes, hypertension, stroke, auto-immune and blood diseases, and eye diseases, as these diseases can cause ocular complications. Participants were categorized as having no disease history, one disease, two diseases, or three or more diseases (multi-morbidity).

### Statistical analysis

We performed a descriptive analysis of all study variables. Statistical significance of the differences between visual status and the participants’ characteristics were determined using the χ2 test. Then, we descriptively examined the differences between the groups that participated with and without visual impairment. Between-group differences were determined using χ2 test. To examine the association between visual status and social participation and subjective health status, we performed a multivariable Poisson regression with a robust variance estimator to calculate the prevalence ratios (PRs) and their 95% confidence intervals (CIs) for poor self-rated health among those with visual impairment (fair/poor) and social participation scores (no participation, one, two, and three or more groups). Then, we performed a multivariable Poisson regression analysis to calculate the PRs and their 95% CIs for poor self-rated health and the social participation score stratified by visual status (with or without visual impairment). For sensitivity analysis, we performed the same analysis among respondents who chose “fair” and “poor” in self-reported visual status to determine if a difference exists between “fair” and “poor” among people with visual impairment. We also examined the interaction between social participation and visual status to examine whether social participation’s association with self-rated health differs according to visual status using data without stratification. A multivariable Poisson regression model with all variables was used to determine statistical significance of the interaction between social participation and visual status. We also conducted a sensitivity analysis using the complete case dataset instead of multiply imputed data.

To account for potential biases due to missing variables, we used multiple imputation techniques. All variables included in the analysis, such as the outcome variable, visual status, explanatory variables, and covariates, were imputed. Under a missing-at-random assumption, we created 10 imputed datasets using a chained equation method, analyzed each dataset, and combined the 10 results using Rubin’s combination method [31]. Following the chained equation method, we performed a logistic regression for the binary variables, a multinomial logistic regression for the categorical variables, and an ordinal logistic regression for the ordinal variables. We treated the comorbidities as binary variables, marital status as a nominal variable, and subjective health, visual status, social participation, total participation score, annual equivalized income, and years of education as ordinal variables. Stata 14 software (StataCorp; College Station, TX) was used to perform the analyses with a 5% significance level.

## Results

**Fig. 1:**
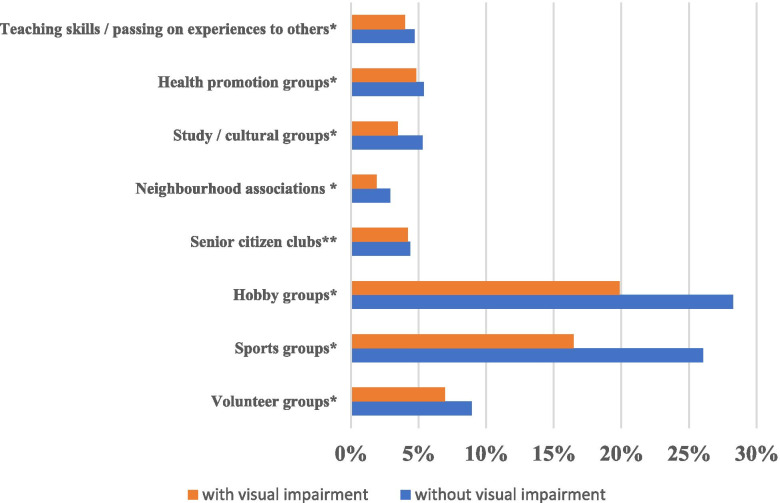
Comparison of social activity type between older adults with and without visual impairment Predictive margins and 95% confidence intervals (CIs) of social participation on poor self-rated health according to visual status. Note. Between-group differences were determined using χ2 test. *p < 0.001, **p = 0.273.

Participants’ mean age was 74.2 (6.3) years (range: 65–100 years); 45.0% were men. Table 1 summarizes the descriptive characteristics according to visual status after multiple imputation. Overall visual impairment prevalence (2063 of 22,291 participants) was 9.3% (95% CI: 8.9–9.7). The percentage of people with visual impairment who reported poor subjective health (38.4%) was approximately three times that of people without visual impairment (13.1%). People with visual impairment were less likely to participate in social activities than those without visual impairment; particularly, they were less likely to be involved in two or more activities. Figure 1 shows the comparison of social activity type between older adults with and without visual impairment. Across all groups, social participation rates were lower for those with visual impairment than for those without (*p* < 0.001), except for senior citizen clubs (*p* = 0.273). The hobby and sports groups had relatively high participation rates for those with visual impairment.

**Table 1:** Descriptive characteristics of study participants by visual status (*N* = 22,291)

	Total		No visual impairment		Visual impairment		
	*N*(%)	% with poor SRH (*N* = 3439)	*N*(%)	% with poor SRH (*N* = 2647)	*N*(%)	% with poor SRH (*N* = 792)	*P* value
**Total**	22,291 (100.0)	15.4	20,228 (100.0)	13.1	2063 (100.0)	38.4	< 0.001
**Age**							
65-69	6621 (29.7)	11.1	6194 (30.6)	9.7	428 (20.7)	31.0	< 0.001
70-74	5906 (26.5)	12.8	5478 (27.1)	11.1	428 (20.7)	34.8	< 0.001
75-79	5057 (22.7)	17.1	4555 (22.5)	14.8	502 (24.3)	38.3	< 0.001
80-84	3171 (14.2)	21.4	2741 (13.5)	17.9	430 (20.9)	44.0	< 0.001
85 or older	1536 (6.9)	26.3	1260 (6.2)	21.8	276 (13.4)	47.0	< 0.001
**Sex**							
Male	10,135 (45.5)	48.6	9206 (45.5)	48.8	929 (45.0)	47.8	0.56
Female	12,156 (54.5)	51.4	11,022 (54.5)	51.2	1134 (55.0)	52.2	0.52
**Marital status**							
Married	16,126 (72.3)	14.6	14,806 (73.2)	12.5	1321 (64.0)	38.1	< 0.001
Widowed	4537 (20.4)	16.8	3995 (19.8)	14.0	542 (26.3)	37.2	< 0.001
Separated	956 (4.3)	18.8	832 (4.1)	15.3	124 (6.0)	42.2	< 0.001
Unmarried	672 (3.0)	21.1	595 (2.9)	17.9	77 (3.7)	46.1	< 0.001
**Education** (years)							
< 9	7480 (33.6)	20.0	6517 (32.2)	16.6	964 (46.7)	42.8	< 0.001
10-12	9177 (41.2)	13.7	8463 (41.8)	12.0	714 (34.6)	34.3	< 0.001
≥ 13	5634 (25.3)	12.2	5249 (25.9)	10.5	386 (18.7)	34.9	< 0.001
**Equivalized income (million yen)**							
Low	11,436 (51.3)	19.0	10,088 (49.9)	15.9	1348 (65.3)	41.7	< 0.001
Middle	8495 (38.1)	12.3	7915 (39.1)	10.8	580 (28.1)	33.3	< 0.001
High	2361 (10.6)	9.4	2225 (11.0)	8.3	136 (6.6)	27.6	< 0.001
**History of systemic comorbidities**							
No history	8684 (39.0)	10.5	8170 (40.4)	9.4	514 (24.9)	28.3	< 0.001
One disease	9626 (43.2)	15.3	8740 (43.2)	13.0	886 (42.9)	37.7	< 0.001
Two diseases	3367 (15.1)	24.1	2853 (14.1)	20.5	513 (24.9)	44.0	< 0.001
Three or more diseases	615 (2.8)	39.4	465 (2.3)	33.4	150 (7.3)	57.8	< 0.001
**Participation in numbers of group**							
No participation	13,615 (61.1)	19.1	12,164 (60.1)	16.2	1452 (70.3)	43.0	< 0.001
1	3025 (13.6)	12.3	2771 (13.7)	10.6	254 (12.3)	30.8	< 0.001
2	3016 (13.5)	8.1	2838 (14.0)	7.3	179 (8.7)	21.2	< 0.001
≥ 3	2635 (11.8)	8.7	2456 (12.1)	7.2	179 (8.7)	28.8	< 0.001

**Fig. 2:**
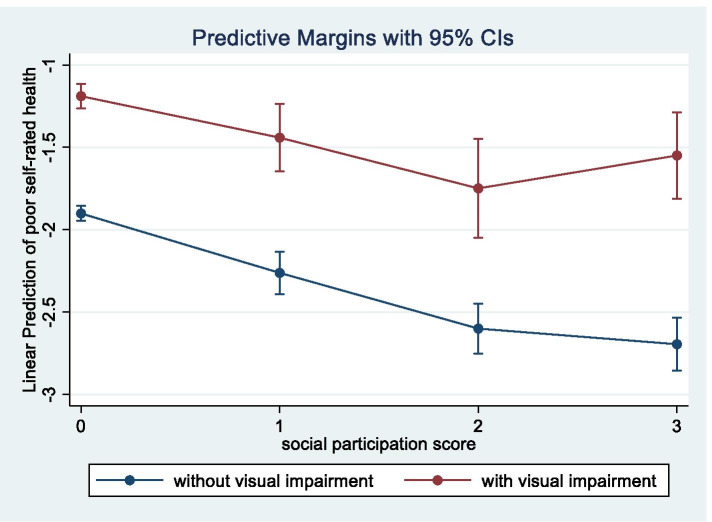
Predictive margins and 95% confidence intervals (CIs) of social participation on poor self-rated health according to visual status. Note. The red line and blue line represent with visual impairment and without visual impairment, respectively. There is no difference in the slope between the two groups when the number of participating groups is two or less

Table 2 shows the results of the multivariable Poisson regression analysis with multiple imputation stratified by visual status. Compared to the group with no visual impairment, the positive impact of social participation on self-rated health was weaker in the visually impaired group, and social participation was significantly associated with poor self-rated health after adjusting for covariates in both groups for one, two, and three or more groups (PR 0.70 vs. 0.76, 0.50 vs.0.54, and 0.45 vs.0.69, respectively). For sensitivity analysis, the “poor” group did not converge in the analysis due to the small sample size, however, the results of the “fair” group (PR 0.78, 0.53, and 0.69, respectively) did not differ significantly from the visually impaired group (“fair” and “poor”). The estimates based on our sensitivity analysis using complete case data did not differ in direction of the result from our original analysis with wider CIs (Supplemental Table). In further interaction analyses for non-stratified data, significant interactions were found only for those who participated in three or more groups (*p* = 0.007). From the model, predictive margins and 95% CIs of social participation on poor self-rated health according to visual status (with or without visual impairment) are shown in Fig. 2. For participation in two or less groups, there was no significant difference in the association between social participation and self-rated health between those with and without visual impairment.

**Table 2:** Prevalence ratios of visual impairment and social participation on poor self-rated health stratified by visual status by multiple logistic regression with multiple imputation

	visual impairment (*n* = 2063)			No visual impairment (*n* = 20,228)		
	Adjusted prevalence ratio*	*P*-value	95% CI	Adjusted prevalence ratio*	*P*-value	95% CI
Participation in numbers of groups						
No participation	reference			reference		
1	0.76	0.013	(0.61-0.94)	0.70	< 0.001	(0.61-0.81)
2	0.54	< 0.001	(0.40-0.74)	0.50	< 0.001	(0.43-0.59)
≥ 3	0.69	0.009	(0.52-0.91)	0.45	< 0.001	(0.38-0.53)

^*^All values are adjusted for other confounders in Table 1

## Discussion

We found that social participation was an important determinant of good self-rated health among community-dwelling Japanese older adults whether they were visually impaired or not. To our knowledge, this is the first interaction analysis conducted between social participation and visual impairment that provides an in-depth examination of the effect of social participation on older adults’ self-rated health.

Considering previous studies, our findings are not surprising. Regarding the relationship between social participation and good self-rated health, a quasi-experimental intervention study found that the odds ratio of participation in senior citizen salon programs for reporting good self-rated health was 2.52 (95% CI: 2.27–2.79) [17]. Generally, social participation reduces the risk of functional disability [19, 20], psychological distress [18], and cognitive impairment [21]. Thus, self-rated health is positively affected by social participation. Additionally, social participation may increase health motivation among older adults because it provides them a space for human interaction and access to various health-related resources, such as instrumental and emotional support and health-relevant information. For example, those with smaller support networks are less likely to receive cataract surgery [32]. Regarding the result that social participation’s positive impact on self-rated health was weaker in the visually impaired population, many prior studies [13, 33, 34] reported a correlation between visual impairment and poor self-rated health. Generally, self-rated health is affected by objective health status, physical/mental disability, and functional limitations; therefore, it is reasonable to assume people with visual impairment report poor self-rated health, considering their limited ability to perform ADLs. Additionally, visual impairment could lead to mortality [12, 35], which is in line with the finding from numerous studies that self-rated health is a predictor of mortality.

On the other hand, the most important finding in the present study was that even if older adults were visually impaired, social participation could have a significant positive impact on their self-rated health: that is, social participation could mitigate the negative association between visual impairment and self-rated health. Regarding this possibility, two studies could be mentioned as follows. While numerous studies have indicated that rates of depression are elevated among the visually impaired [36], significant differences in depression scores were not found between adolescents without visual impairment and adolescents with visual impairment who attend a specialized school for the blind [37] or Finnish regular schools [38]. Because one of the roles of these schools for adolescents with visual impairment is to improve the self-value of the person and to facilitate social interaction, the results above might be valid. Therefore, it is to be desired that the value of social participation for older adults with visual impairment would approach that of these specialized schools for adolescents with visual impairment.

Our present findings emphasize the importance of encouraging older adults to participate in several social activities, whether they have visual impairment or not, to potentially reduce their risk of experiencing worsening general health. It is possible for social activity-related interventions to be advanced because social participation has always been promoted as a way of maintaining functional independence and healthy aging [17]. To encourage older adults with visual impairment to increase their social activities, we need to better understand the factors contributing to their participation intention. To clarify these factors, a content analysis was conducted with 21 individuals with impaired vision, revealing that stigma and stereotyping experienced by older adults and difficult environmental contexts were barriers to social participation [39]. Strategies for reduced prejudice might therefore increase social participation. Other factors associated with the increased social participation of 364 older adults with severely impaired vision were better income level and higher attachment to their neighborhood [40].

The present study revealed that Japanese older adults participated in hobby and sports groups more frequently, regardless of visual status, though participation rates were lower for those with visual impairment across all social activity types. Further, visual impairment had a larger negative effect for these two social activities than for other social activity types. Therefore, encouraging older adults to participate in hobby and sports groups for their general health is reasonable, and special care should be taken to ensure that older adults with visual impairment participate equally. As described in Fig. 1, on the other hand, the social participation score ≥ 3 in adults with visual impairment did not lead to expected gradual improvement of self-rated health compared to older adults without visual impairment. This result implies that frequent social participation might not lead to greater improvement of self-rated health for older adults with visual impairment.

The Centers for Disease Control and Prevention (CDC), which recommends a minimum of 150 min of moderate activity per week, emphasizes there is strong evidence that regular physical activity creates major health benefits for individuals with a disability [41]. In the USA, disabilities in mobility, cognition, independent living, hearing and vision are the five most common functional disabilities [42]. On the other hand, individuals with visual impairment have been reported to have a fear of falling due to low visual function, and therefore, to spend less time engaged in moderate–vigorous physical activity (26–48%) compared to individuals without visual impairment [43, 44]. In many cases, in fact, visual impairment is the exclusion factor for physical activity interventions [45]. However, a systematic review [45] illustrated that physical activity interventions for adults with visual impairment, such as Tai chi, Yoga, and dance, could have positive results, particularly in physical measures such as mobility and balance. More importantly, the review revealed that no paper reporting negative results found the intervention to be detrimental to any aspect of health measured. This result may support health workers to encourage older adults with visual impairment to participate in these social activities. More research is required into the respective effectiveness of the social activities on self-rated health, in order to understand which social activities are most rational and suitable for older adults with visual impairment.

A major strength of this study is that we used a large population-based dataset. The large sample size permitted us to identify 2063 older adults with visual impairment and allowed for the large-scale visual status-stratified analysis of the association between social participation and self-rated health. We adjusted for the following possible confounding factors: age, sex, marital status, educational level, annual equivalized income level, and history of systemic comorbidities (Table 1). Our study has several limitations. First, as the study was cross-sectional, we could not exclude the possibility of reverse causation. While social participation affects self-rated health, self-rated health could influence social participation. Previous studies indicated older adults with poor self-rated health were less likely to be very active or to exercise [28] and that older adults with better self-rated health were more likely to engage in self-care, controlling for chronic conditions, symptoms, and psychosocial factors [46]. Without longitudinal study, it is not possible to establish a true causal relationship. Second, this study used self-reported vision status instead of objective measures. Although self-reported vision status is widely used in epidemiologic research, and its validity has been confirmed, it is not equivalent to objective testing of visual acuity [47, 48]. Conversely, self-reported vision status reflects broad aspects of vision that directly affect older adults’ lives under non-ideal conditions, such as low contrast, glare, and low and changing levels of light [49]. Thus, a self-reported measure may reflect vision with regard to everyday functioning more accurately. Third, the sample analyzed cannot be considered representative of all older adults, as the institutionalized population was not included. While the present study revealed the association between social participation and good self-rated health, future work would benefit from longitudinal analyses that use objective measures of vision and include not only the data of community dwelling older adults but also that of institutionalized older adults.

In conclusion, social participation was strongly associated with good self-rated health even if the subjects were older adults with visual impairment. Encouraging older adults, regardless of visual impairment status, to participate in social activities could lead to improvement in their self-rated health.

## Supplementary Information

**Additional file 1: Supplemental Table.** Prevalence ratios of visual impairment and social participation on poor self-rated health stratified by visual status by complete case dataset.

## Data Availability

The datasets of the Japan Gerontological Evaluation Study, which were used in this research, are available from the corresponding author upon reasonable request. All enquiries should be addressed to the data management committee via e-mail: dataadmin.ml@jages.net. All JAGES datasets have ethical or legal restrictions for public deposition due to the inclusion of sensitive information from the human participants. Following the regulations of local governments which cooperated in the survey, the JAGES data management committee has imposed restrictions upon the data.
