# Supplementary material for: Is social participation associated with good self-rated health among visually impaired older adults?: the JAGES cross-sectional study
Source: BMC Geriatr. 2021 Oct 23;21:592. doi: 10.1186/s12877-021-02554-7 (PMC8539799; doi:10.1186/s12877-021-02554-7)
Supplement: Supplementary file 1 — Additional file 1: Supplemental Table. Prevalence ratios of visual impairment and social participation on poor self-rated health stratified by visual status by complete case dataset. [file 12877_2021_2554_MOESM1_ESM.docx]

Supplemental Table. Prevalence ratios of visual impairment and social participation on poor self-rated health stratified by visual status by complete case dataset.

|  | visual impairment (*n*=1,180) | | |  | No visual impairment (*n*=13,378) | | |
| --- | --- | --- | --- | --- | --- | --- | --- |
|  | Adjusted prevalence ratio* | *P*-value | 95% CI |  | Adjusted prevalence ratio* | *P*-value | 95% CI |
| Participation in numbers of groups |  |  |  |  |  |  |  |
| no participation | reference |  |  |  | reference |  |  |
| 1 | 0.81 | 0.192 | (0.59-1.11) |  | 0.69 | <0.001 | (0.60-0.80) |
| 2 | 0.50 | 0.005 | (0.31-0.81) |  | 0.51 | <0.001 | (0.42-0.61) |
| ≥3 | 0.63 | 0.119 | (0.35-1.13) |  | 0.47 | <0.001 | (0.37-0.60) |

CI, confidence interval.

^a^All values are adjusted for other confounders in Table 1.
